# 8-(2-Chloro­phen­yl)-1-(4-chloro­phen­yl)-4-[(*E*)-(2-chloro­phen­yl)methyl­idene]-6-methyl-4,5,6,7,7a,8-hexa­hydro-1,2,4-oxadiazolo[5,4-*d*]pyrido[3,4-*c*][1,5]benzothia­zepine

**DOI:** 10.1107/S1600536810013309

**Published:** 2010-04-21

**Authors:** V. Rajni Swamy, R. Sudha Periathai, K. Rajagopal, R. V. Krishnakumar, N. Srinivasan

**Affiliations:** a Department of Physics, Thiagarajar College, Madurai 625 009, India; b Department of Physics, The Standard Fireworks Rajaratnam College for Women, Sivakasi 626 123, India; c Department of Physics, Saraswathy Narayanan College, Madurai 625 022, India

## Abstract

In the title compound, C_33_H_26_Cl_3_N_3_OS, the oxadiazole, piperidine and benzothia­pezine rings adopt envelope, chair and twist-boat conformations, respectively. In the crystal, the mol­ecular aggregation is characterized by chains of centrosymmetrically related pairs connected through Cl⋯Cl inter­actions [3.533 (2) Å], extending parallel to (202).

## Related literature

For the biological importance of benziothia­zepines and oxadiazol derivatives, see: Budriesi *et al.* (2007 ▶); Sahin *et al.* (2002 ▶). For ring geometry, see: Boeyens (1978 ▶); Cremer & Pople (1975 ▶). For a related structure, see: Srinivasan *et al.* (2007 ▶).
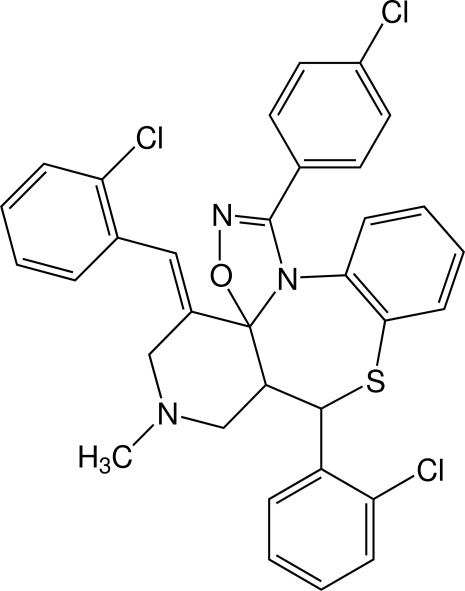


## Experimental

### 

#### Crystal data

C_33_H_26_Cl_3_N_3_OS
*M* *_r_* = 618.98
Triclinic, 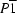

*a* = 11.015 (3) Å
*b* = 11.758 (4) Å
*c* = 11.988 (4) Å
α = 78.87 (2)°
β = 86.89 (3)°
γ = 78.29 (2)°
*V* = 1491.5 (8) Å^3^
*Z* = 2
Mo *K*α radiation
μ = 0.41 mm^−1^
*T* = 298 K
0.30 × 0.15 × 0.15 mm

#### Data collection

Bruker Kappa APEXII CCD diffractometer
Absorption correction: multi-scan (*SADABS*; Sheldrick, 2004 ▶) *T*_min_ = 0.90, *T*_max_ = 0.94
42055 measured reflections
11096 independent reflections
7867 reflections with *I* > 2σ(*I*)
*R*_int_ = 0.023

#### Refinement

*R*[*F* ^2^ > 2σ(*F* ^2^)] = 0.048
*wR*(*F* ^2^) = 0.146
*S* = 1.03
11096 reflections
372 parameters
H-atom parameters constrained
Δρ_max_ = 0.64 e Å^−3^
Δρ_min_ = −0.62 e Å^−3^

### 

Data collection: *APEX2* (Bruker, 2004 ▶); cell refinement: *SAINT-Plus* (Bruker, 2004 ▶); data reduction: *SAINT-Plus*; program(s) used to solve structure: *SHELXS97* (Sheldrick, 2008 ▶); program(s) used to refine structure: *SHELXL97* (Sheldrick, 2008 ▶); molecular graphics: *PLATON* (Spek, 2009 ▶); software used to prepare material for publication: *SHELXL97*.

## Supplementary Material

Crystal structure: contains datablocks I, global. DOI: 10.1107/S1600536810013309/nc2178sup1.cif

Structure factors: contains datablocks I. DOI: 10.1107/S1600536810013309/nc2178Isup2.hkl

Additional supplementary materials:  crystallographic information; 3D view; checkCIF report

## Figures and Tables

**Fig. 1.**
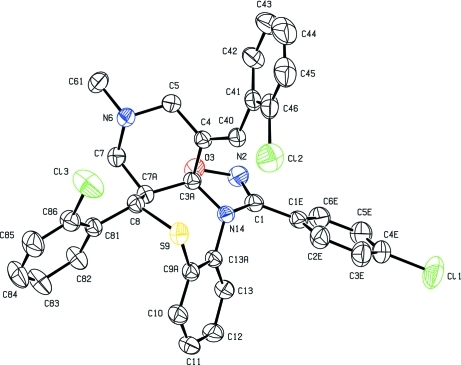
The molecular structure of (I), with atom labels and 50% probability displacement ellipsoids for non-H atoms. H atoms have been omitted for clarity.

**Fig. 2.**
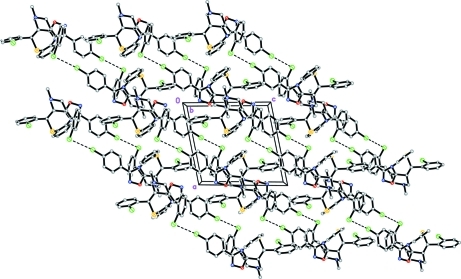
A view of the molecular aggregation down the a--axis. H atoms have been omitted and Cl···Cl interactions are indicated by dashed lines.

## supplementary crystallographic information

### Comment

The title compound, C_33_H_26_N_3_OCl_3_ S, belongs to an important class of heterocycles which exhibit antihypertensive properties. The compound consists of a benzothiazepine, a oxadiazole and a methyl piperidine ring. Benziothiazepines are regarded as a class of calcium channel blockers (Budriesi *et al.*, 2007), oxadiazol derivatives are established as micobicides (Sahin *et al.*, 2002) and piperidines are established as key components of anti-Parkinson's drugs. Accurate description of the molecular geometry of such molecules are indispensable to proceed with the pharmacological investigations which may prove useful in the design of drugs with a wide range of activities. Also, the role of non-conventional hydrogen bonds viz. C—H···X (X= N, O, Cl, F, etc.) in influencing the geometry of the molecular packing can be unambiguously assessed. Recently, an anlogue of the title compound namely, the crystal structure of 1-(4-chlorophenyl)- 8-(4-fluorophenyl)-4-[(*E*)-(4-fluorophenyl)methylidene]-6-methyl-4,5,6,7,7a, 8-hexahydro[1,2,4]oxadiazolo[5,4-d]pyrido [3,4-c][1,5] benzothiazepine (Srinivasan *et al.*, 2007) was elucidated.

A least-squares plane calculations show that the 2-chlorophenyl attached to thiazepine, 2-chlorophenyl attached to piperidine and 4-chlorophenyl ring of the oxadiazole ring make a dihedral angle of 34.8 (1) °, 51.3 (1) ° and 73.9 (1) °, respectively, with respect to the benzene fused to the thiazepine ring. The torsion angles about the methylidene bond C4—C40—C41—C42 = 39.8 (2) ° and C4—C40—C41—C46 = -141.8 (2) ° indicates a significant twist of the 2-chlorophenyl ring which may be attributed to steric factors. These values that describe the molecular geometry slightly differ from those observed in the 4-fluoro-4-fluoro- 4-chloro analogue (Srinivasan *et al.*, 2007). The oxadiazole, piperidine and benzothiapezine rings adopt the usually expected envelope, chair and twist-boat conformations, respectively. The molecular aggreagation is characterized by linear chains of centrosymmetrically related pairs extending parallel to the (202) plane and connected through Cl···Cl interactions [Cl1···Cl2(-x+1,-y,-z+2) = 3.533 (2) Å. Other Cl···Cl distances observed are Cl2···Cl2(-x+1,-y+1,-z+1) = 3.826 (2) Å and Cl1···Cl3(x,+y-1,+z+1) = 3.952 (2) Å.

### Experimental

2-Methyl-11-(2-chlorophenyl)-4-[(*E*)-(2-chlorophenyl)methylidene]- 1,2,3,4,11,11a-hexahydro-pyrido[3,4-c][1,5]benzothiazepines (1 mmol) and 4-chloro-*N*-hydroxybenzenecarboximidoyl chloride (1 mmol) were dissolved in benzene (15 ml). Triethylamine (1 mmol) was added to the mixture and refluxed for 20 to 30 min. After completion of the reaction as evident from thin layer chromatography the triethylamine hydrochloride was filtered off, solvent evaporated, product was purified by column chromatography using petroleum ether:ethyl acetate (90:10 v/v) mixture and finally recrystallized from ethyl acetate to obtain pure 1-(4-chlorophenyl)-8-(2-chlorophenyl) -4-[(*E*)-(2-chlorophenyl)methylidene]-6-methyl-4,5,6,7,7a,8- hexahydro[1,2,4]oxa-diazolo[5,4-d]pyrido[3,4-c][1,5] benzothiazepine as colorless crystals.

### Refinement

H atoms were positioned geometrically and refined using a riding model with C—H = 0.95–0.99 Å and with *U*_iso_(H) = 1.2 (1.5 for methyl groups) times *U*_eq_(C).

### Crystal data

**Table:**

C_33_H_26_Cl_3_N_3_OS	*Z* = 2
*M**_r_* = 618.98	*F*(000) = 640
Triclinic, *P*1	*D*_x_ = 1.378 Mg m^−^^3^
Hall symbol: -P 1	Mo *K*α radiation, λ = 0.71073 Å
*a* = 11.015 (3) Å	Cell parameters from 4125 reflections
*b* = 11.758 (4) Å	θ = 2.0–30.0°
*c* = 11.988 (4) Å	µ = 0.41 mm^−^^1^
α = 78.87 (2)°	*T* = 298 K
β = 86.89 (3)°	Needle, colourless
γ = 78.29 (2)°	0.30 × 0.15 × 0.15 mm
*V* = 1491.5 (8) Å^3^	

### Data collection

**Table:**

Bruker Kappa APEXII CCD diffractometer	11096 independent reflections
Radiation source: fine-focus sealed tube	7867 reflections with *I* > 2σ(*I*)
graphite	*R*_int_ = 0.023
ω and φ scan	θ_max_ = 33.0°, θ_min_ = 1.7°
Absorption correction: multi-scan (*SADABS*; Sheldrick, 2004)	*h* = −16→16
*T*_min_ = 0.90, *T*_max_ = 0.94	*k* = −17→17
42055 measured reflections	*l* = −18→18

### Refinement

**Table:**

Refinement on *F*^2^	Primary atom site location: structure-invariant direct methods
Least-squares matrix: full	Secondary atom site location: difference Fourier map
*R*[*F*^2^ > 2σ(*F*^2^)] = 0.048	Hydrogen site location: inferred from neighbouring sites
*wR*(*F*^2^) = 0.146	H-atom parameters constrained
*S* = 1.03	*w* = 1/[σ^2^(*F*_o_^2^) + (0.0605*P*)^2^ + 0.6302*P*] where *P* = (*F*_o_^2^ + 2*F*_c_^2^)/3
11096 reflections	(Δ/σ)_max_ = 0.001
372 parameters	Δρ_max_ = 0.64 e Å^−^^3^
0 restraints	Δρ_min_ = −0.62 e Å^−^^3^

### Special details

**Table:**

Geometry. All esds (except the esd in the dihedral angle between two l.s. planes) are estimated using the full covariance matrix. The cell esds are taken into account individually in the estimation of esds in distances, angles and torsion angles; correlations between esds in cell parameters are only used when they are defined by crystal symmetry. An approximate (isotropic) treatment of cell esds is used for estimating esds involving l.s. planes.
Refinement. Refinement of *F*^2^ against ALL reflections. The weighted *R*-factor wR and goodness of fit *S* are based on *F*^2^, conventional *R*-factors *R* are based on *F*, with *F* set to zero for negative *F*^2^. The threshold expression of *F*^2^ > σ(*F*^2^) is used only for calculating *R*-factors(gt) etc. and is not relevant to the choice of reflections for refinement. *R*-factors based on *F*^2^ are statistically about twice as large as those based on *F*, and *R*- factors based on ALL data will be even larger.

### Fractional atomic coordinates and isotropic or equivalent isotropic displacement parameters (Å^2^)

**Table:**

	*x*	*y*	*z*	*U*_iso_*/*U*_eq_	
Cl1	0.57470 (7)	−0.12556 (6)	1.22882 (7)	0.0878 (2)	
Cl2	0.55422 (5)	0.34566 (6)	0.58843 (6)	0.07294 (17)	
Cl3	0.71901 (7)	0.77874 (5)	0.53545 (4)	0.0769 (2)	
C1	0.85278 (14)	0.22540 (11)	0.96401 (12)	0.0336 (3)	
N2	0.97082 (12)	0.20144 (11)	0.95229 (12)	0.0404 (3)	
O3	1.00715 (9)	0.30577 (9)	0.89247 (9)	0.0378 (2)	
C3A	0.89581 (12)	0.37735 (11)	0.83332 (11)	0.0300 (2)	
C4	0.89945 (13)	0.33994 (11)	0.71870 (12)	0.0320 (3)	
C5	1.01522 (14)	0.36089 (13)	0.65093 (13)	0.0368 (3)	
H5A	1.0857	0.3030	0.6843	0.044*	
H5B	1.0069	0.3495	0.5739	0.044*	
N6	1.03901 (12)	0.48001 (11)	0.64726 (10)	0.0353 (2)	
C7	1.03493 (13)	0.51369 (13)	0.75868 (13)	0.0355 (3)	
H7A	1.0489	0.5938	0.7497	0.043*	
H7B	1.1005	0.4617	0.8053	0.043*	
C7A	0.90965 (12)	0.50621 (11)	0.81809 (12)	0.0307 (2)	
H71A	0.9104	0.5276	0.8931	0.037*	
C8	0.80644 (13)	0.59400 (11)	0.74674 (12)	0.0315 (2)	
H8	0.8252	0.5869	0.6672	0.038*	
S9	0.65096 (3)	0.56089 (3)	0.77852 (3)	0.03747 (9)	
C9A	0.65984 (13)	0.52634 (12)	0.92758 (13)	0.0333 (3)	
C10	0.59577 (15)	0.60462 (13)	0.99383 (16)	0.0439 (3)	
H10	0.5521	0.6780	0.9589	0.053*	
C11	0.59637 (17)	0.57444 (16)	1.11118 (17)	0.0484 (4)	
H11	0.5521	0.6266	1.1549	0.058*	
C12	0.66289 (16)	0.46670 (16)	1.16287 (14)	0.0437 (3)	
H12	0.6613	0.4453	1.2417	0.052*	
C13	0.73233 (14)	0.38968 (13)	1.09826 (13)	0.0368 (3)	
H13	0.7803	0.3188	1.1340	0.044*	
C13A	0.73021 (12)	0.41847 (11)	0.98022 (12)	0.0303 (2)	
N14	0.79641 (11)	0.34047 (9)	0.91010 (10)	0.0300 (2)	
C61	1.15951 (16)	0.48759 (16)	0.59264 (15)	0.0462 (4)	
H61A	1.1759	0.5652	0.5899	0.069*	
H61B	1.1591	0.4726	0.5168	0.069*	
H61C	1.2229	0.4299	0.6354	0.069*	
C1E	0.78313 (14)	0.13884 (11)	1.02695 (12)	0.0350 (3)	
C2E	0.65779 (16)	0.15047 (14)	1.01121 (16)	0.0458 (4)	
H2E	0.6168	0.2127	0.9579	0.055*	
C3E	0.59237 (19)	0.07021 (16)	1.07412 (19)	0.0555 (5)	
H3E	0.5074	0.0796	1.0650	0.067*	
C4E	0.6548 (2)	−0.02356 (15)	1.15020 (17)	0.0527 (4)	
C5E	0.7800 (2)	−0.03845 (15)	1.16498 (16)	0.0529 (4)	
H5E	0.8211	−0.1032	1.2156	0.063*	
C6E	0.84464 (17)	0.04309 (13)	1.10441 (14)	0.0443 (3)	
H6E	0.9293	0.0341	1.1153	0.053*	
C40	0.80948 (15)	0.29252 (12)	0.68793 (12)	0.0364 (3)	
H40	0.7441	0.2867	0.7398	0.044*	
C41	0.79968 (17)	0.24807 (13)	0.58271 (13)	0.0419 (3)	
C42	0.9013 (2)	0.18426 (17)	0.53161 (17)	0.0557 (5)	
H42	0.9788	0.1682	0.5647	0.067*	
C43	0.8879 (3)	0.1446 (2)	0.4319 (2)	0.0757 (7)	
H43	0.9568	0.1037	0.3980	0.091*	
C44	0.7738 (3)	0.1654 (2)	0.38322 (19)	0.0815 (8)	
H44	0.7656	0.1387	0.3164	0.098*	
C45	0.6718 (3)	0.2253 (2)	0.43257 (18)	0.0690 (6)	
H45	0.5942	0.2383	0.4002	0.083*	
C46	0.68514 (19)	0.26646 (16)	0.53113 (15)	0.0503 (4)	
C81	0.80119 (13)	0.72208 (12)	0.75331 (12)	0.0336 (3)	
C82	0.82787 (18)	0.75779 (14)	0.85131 (15)	0.0460 (4)	
H82	0.8572	0.7006	0.9140	0.055*	
C83	0.8122 (2)	0.87629 (16)	0.85885 (19)	0.0585 (5)	
H83	0.8297	0.8974	0.9262	0.070*	
C84	0.7708 (2)	0.96217 (16)	0.7669 (2)	0.0635 (5)	
H84	0.7607	1.0416	0.7717	0.076*	
C85	0.7445 (2)	0.93064 (15)	0.66774 (18)	0.0598 (5)	
H85	0.7168	0.9885	0.6050	0.072*	
C86	0.75923 (17)	0.81224 (14)	0.66168 (14)	0.0436 (3)	

### Atomic displacement parameters (Å^2^)

**Table:**

	*U*^11^	*U*^22^	*U*^33^	*U*^12^	*U*^13^	*U*^23^
Cl1	0.1016 (5)	0.0606 (3)	0.0984 (5)	−0.0388 (3)	0.0198 (4)	0.0104 (3)
Cl2	0.0590 (3)	0.0861 (4)	0.0774 (4)	−0.0188 (3)	−0.0099 (3)	−0.0169 (3)
Cl3	0.1281 (5)	0.0532 (3)	0.0411 (2)	0.0047 (3)	−0.0262 (3)	−0.0051 (2)
C1	0.0396 (7)	0.0255 (5)	0.0314 (6)	0.0003 (5)	0.0013 (5)	−0.0025 (5)
N2	0.0403 (6)	0.0324 (6)	0.0411 (7)	0.0011 (5)	0.0000 (5)	0.0022 (5)
O3	0.0318 (5)	0.0364 (5)	0.0399 (5)	−0.0018 (4)	−0.0024 (4)	0.0015 (4)
C3A	0.0312 (6)	0.0271 (5)	0.0302 (6)	−0.0029 (4)	−0.0011 (5)	−0.0043 (4)
C4	0.0377 (7)	0.0256 (5)	0.0318 (6)	−0.0046 (5)	0.0018 (5)	−0.0054 (4)
C5	0.0399 (7)	0.0343 (6)	0.0366 (7)	−0.0070 (5)	0.0049 (6)	−0.0091 (5)
N6	0.0375 (6)	0.0350 (6)	0.0335 (6)	−0.0097 (5)	0.0018 (5)	−0.0044 (4)
C7	0.0357 (7)	0.0349 (6)	0.0371 (7)	−0.0096 (5)	−0.0016 (5)	−0.0067 (5)
C7A	0.0334 (6)	0.0278 (5)	0.0314 (6)	−0.0065 (5)	−0.0025 (5)	−0.0055 (5)
C8	0.0366 (6)	0.0276 (5)	0.0303 (6)	−0.0060 (5)	−0.0027 (5)	−0.0048 (4)
S9	0.03504 (17)	0.03241 (16)	0.0429 (2)	−0.00581 (13)	−0.00816 (14)	−0.00064 (13)
C9A	0.0302 (6)	0.0275 (6)	0.0415 (7)	−0.0045 (5)	0.0010 (5)	−0.0063 (5)
C10	0.0401 (8)	0.0306 (6)	0.0599 (10)	−0.0017 (6)	0.0067 (7)	−0.0133 (6)
C11	0.0483 (9)	0.0447 (8)	0.0581 (10)	−0.0099 (7)	0.0122 (8)	−0.0266 (8)
C12	0.0472 (8)	0.0509 (9)	0.0387 (8)	−0.0146 (7)	0.0045 (6)	−0.0187 (7)
C13	0.0387 (7)	0.0368 (7)	0.0357 (7)	−0.0069 (5)	−0.0009 (5)	−0.0092 (5)
C13A	0.0294 (6)	0.0271 (5)	0.0349 (6)	−0.0046 (4)	0.0007 (5)	−0.0080 (5)
N14	0.0330 (5)	0.0231 (4)	0.0310 (5)	−0.0016 (4)	0.0021 (4)	−0.0031 (4)
C61	0.0456 (8)	0.0512 (9)	0.0429 (8)	−0.0179 (7)	0.0091 (7)	−0.0052 (7)
C1E	0.0426 (7)	0.0238 (5)	0.0351 (7)	−0.0006 (5)	0.0046 (5)	−0.0046 (5)
C2E	0.0457 (8)	0.0315 (7)	0.0553 (10)	−0.0037 (6)	−0.0005 (7)	−0.0006 (6)
C3E	0.0514 (10)	0.0409 (8)	0.0724 (13)	−0.0125 (7)	0.0056 (9)	−0.0042 (8)
C4E	0.0684 (12)	0.0340 (7)	0.0553 (10)	−0.0164 (7)	0.0136 (9)	−0.0042 (7)
C5E	0.0704 (12)	0.0319 (7)	0.0474 (9)	−0.0029 (7)	0.0042 (8)	0.0057 (6)
C6E	0.0503 (9)	0.0322 (7)	0.0434 (8)	0.0006 (6)	0.0012 (7)	0.0006 (6)
C40	0.0456 (8)	0.0308 (6)	0.0342 (7)	−0.0119 (5)	0.0033 (6)	−0.0062 (5)
C41	0.0631 (10)	0.0334 (7)	0.0341 (7)	−0.0221 (7)	0.0032 (7)	−0.0061 (5)
C42	0.0753 (13)	0.0449 (9)	0.0521 (10)	−0.0158 (9)	0.0077 (9)	−0.0201 (8)
C43	0.118 (2)	0.0592 (12)	0.0582 (13)	−0.0252 (13)	0.0204 (14)	−0.0288 (10)
C44	0.141 (3)	0.0757 (15)	0.0429 (11)	−0.0470 (17)	0.0015 (14)	−0.0220 (10)
C45	0.1051 (18)	0.0694 (13)	0.0436 (10)	−0.0437 (13)	−0.0145 (11)	−0.0061 (9)
C46	0.0713 (12)	0.0456 (8)	0.0402 (8)	−0.0299 (8)	−0.0033 (8)	−0.0028 (7)
C81	0.0385 (7)	0.0273 (5)	0.0346 (7)	−0.0064 (5)	−0.0007 (5)	−0.0051 (5)
C82	0.0612 (10)	0.0329 (7)	0.0447 (8)	−0.0057 (7)	−0.0120 (7)	−0.0100 (6)
C83	0.0764 (13)	0.0389 (8)	0.0653 (12)	−0.0070 (8)	−0.0167 (10)	−0.0223 (8)
C84	0.0848 (15)	0.0293 (7)	0.0776 (14)	−0.0098 (8)	−0.0056 (11)	−0.0131 (8)
C85	0.0850 (14)	0.0296 (7)	0.0580 (11)	−0.0053 (8)	−0.0037 (10)	0.0032 (7)
C86	0.0584 (10)	0.0328 (7)	0.0365 (7)	−0.0057 (6)	−0.0010 (7)	−0.0022 (5)

### Geometric parameters (Å, °)

**Table:**

Cl1—C4E	1.7345 (19)	C13A—N14	1.4279 (17)
Cl2—C46	1.736 (2)	C61—H61A	0.9600
Cl3—C86	1.7364 (19)	C61—H61B	0.9600
C1—N2	1.279 (2)	C61—H61C	0.9600
C1—N14	1.4138 (17)	C1E—C2E	1.379 (2)
C1—C1E	1.467 (2)	C1E—C6E	1.394 (2)
N2—O3	1.4169 (17)	C2E—C3E	1.386 (3)
O3—C3A	1.4698 (17)	C2E—H2E	0.9300
C3A—N14	1.4708 (18)	C3E—C4E	1.376 (3)
C3A—C4	1.518 (2)	C3E—H3E	0.9300
C3A—C7A	1.5285 (19)	C4E—C5E	1.371 (3)
C4—C40	1.331 (2)	C5E—C6E	1.381 (2)
C4—C5	1.511 (2)	C5E—H5E	0.9300
C5—N6	1.4684 (19)	C6E—H6E	0.9300
C5—H5A	0.9700	C40—C41	1.472 (2)
C5—H5B	0.9700	C40—H40	0.9300
N6—C61	1.459 (2)	C41—C46	1.394 (3)
N6—C7	1.461 (2)	C41—C42	1.398 (3)
C7—C7A	1.529 (2)	C42—C43	1.389 (3)
C7—H7A	0.9700	C42—H42	0.9300
C7—H7B	0.9700	C43—C44	1.372 (4)
C7A—C8	1.5420 (19)	C43—H43	0.9300
C7A—H71A	0.9800	C44—C45	1.370 (4)
C8—C81	1.5124 (19)	C44—H44	0.9300
C8—S9	1.8364 (16)	C45—C46	1.386 (3)
C8—H8	0.9800	C45—H45	0.9300
S9—C9A	1.7580 (17)	C81—C82	1.386 (2)
C9A—C10	1.391 (2)	C81—C86	1.397 (2)
C9A—C13A	1.3974 (19)	C82—C83	1.389 (2)
C10—C11	1.383 (3)	C82—H82	0.9300
C10—H10	0.9300	C83—C84	1.371 (3)
C11—C12	1.377 (3)	C83—H83	0.9300
C11—H11	0.9300	C84—C85	1.373 (3)
C12—C13	1.389 (2)	C84—H84	0.9300
C12—H12	0.9300	C85—C86	1.384 (2)
C13—C13A	1.390 (2)	C85—H85	0.9300
C13—H13	0.9300		
			
N2—C1—N14	114.41 (13)	N6—C61—H61A	109.5
N2—C1—C1E	122.05 (12)	N6—C61—H61B	109.5
N14—C1—C1E	123.53 (13)	H61A—C61—H61B	109.5
C1—N2—O3	106.95 (11)	N6—C61—H61C	109.5
N2—O3—C3A	105.63 (10)	H61A—C61—H61C	109.5
O3—C3A—N14	101.63 (10)	H61B—C61—H61C	109.5
O3—C3A—C4	105.55 (10)	C2E—C1E—C6E	119.32 (15)
N14—C3A—C4	113.86 (11)	C2E—C1E—C1	121.26 (13)
O3—C3A—C7A	106.44 (11)	C6E—C1E—C1	119.43 (15)
N14—C3A—C7A	117.54 (11)	C1E—C2E—C3E	120.60 (16)
C4—C3A—C7A	110.41 (11)	C1E—C2E—H2E	119.7
C40—C4—C5	126.87 (13)	C3E—C2E—H2E	119.7
C40—C4—C3A	121.26 (13)	C4E—C3E—C2E	119.13 (19)
C5—C4—C3A	111.85 (12)	C4E—C3E—H3E	120.4
N6—C5—C4	112.64 (11)	C2E—C3E—H3E	120.4
N6—C5—H5A	109.1	C5E—C4E—C3E	121.18 (16)
C4—C5—H5A	109.1	C5E—C4E—Cl1	119.05 (15)
N6—C5—H5B	109.1	C3E—C4E—Cl1	119.77 (17)
C4—C5—H5B	109.1	C4E—C5E—C6E	119.72 (16)
H5A—C5—H5B	107.8	C4E—C5E—H5E	120.1
C61—N6—C7	109.80 (12)	C6E—C5E—H5E	120.1
C61—N6—C5	108.88 (12)	C5E—C6E—C1E	120.02 (17)
C7—N6—C5	113.90 (11)	C5E—C6E—H6E	120.0
N6—C7—C7A	110.94 (12)	C1E—C6E—H6E	120.0
N6—C7—H7A	109.5	C4—C40—C41	128.64 (14)
C7A—C7—H7A	109.5	C4—C40—H40	115.7
N6—C7—H7B	109.5	C41—C40—H40	115.7
C7A—C7—H7B	109.5	C46—C41—C42	117.06 (16)
H7A—C7—H7B	108.0	C46—C41—C40	120.18 (16)
C3A—C7A—C7	106.66 (11)	C42—C41—C40	122.74 (17)
C3A—C7A—C8	113.68 (11)	C43—C42—C41	120.8 (2)
C7—C7A—C8	109.23 (12)	C43—C42—H42	119.6
C3A—C7A—H71A	109.1	C41—C42—H42	119.6
C7—C7A—H71A	109.1	C44—C43—C42	120.4 (2)
C8—C7A—H71A	109.1	C44—C43—H43	119.8
C81—C8—C7A	114.25 (11)	C42—C43—H43	119.8
C81—C8—S9	108.47 (10)	C45—C44—C43	120.3 (2)
C7A—C8—S9	113.89 (10)	C45—C44—H44	119.9
C81—C8—H8	106.6	C43—C44—H44	119.9
C7A—C8—H8	106.6	C44—C45—C46	119.5 (2)
S9—C8—H8	106.6	C44—C45—H45	120.3
C9A—S9—C8	98.49 (6)	C46—C45—H45	120.3
C10—C9A—C13A	119.60 (14)	C45—C46—C41	122.0 (2)
C10—C9A—S9	120.58 (12)	C45—C46—Cl2	117.89 (18)
C13A—C9A—S9	119.82 (11)	C41—C46—Cl2	120.09 (14)
C11—C10—C9A	120.63 (15)	C82—C81—C86	116.15 (14)
C11—C10—H10	119.7	C82—C81—C8	122.89 (13)
C9A—C10—H10	119.7	C86—C81—C8	120.79 (13)
C12—C11—C10	119.64 (15)	C81—C82—C83	122.09 (16)
C12—C11—H11	120.2	C81—C82—H82	119.0
C10—C11—H11	120.2	C83—C82—H82	119.0
C11—C12—C13	120.55 (16)	C84—C83—C82	119.96 (18)
C11—C12—H12	119.7	C84—C83—H83	120.0
C13—C12—H12	119.7	C82—C83—H83	120.0
C12—C13—C13A	120.05 (14)	C83—C84—C85	119.84 (17)
C12—C13—H13	120.0	C83—C84—H84	120.1
C13A—C13—H13	120.0	C85—C84—H84	120.1
C13—C13A—C9A	119.41 (13)	C84—C85—C86	119.65 (17)
C13—C13A—N14	122.17 (12)	C84—C85—H85	120.2
C9A—C13A—N14	118.41 (13)	C86—C85—H85	120.2
C1—N14—C13A	117.65 (11)	C85—C86—C81	122.31 (16)
C1—N14—C3A	102.39 (10)	C85—C86—Cl3	117.21 (14)
C13A—N14—C3A	119.70 (11)	C81—C86—Cl3	120.46 (12)
			
N14—C1—N2—O3	3.72 (17)	C9A—C13A—N14—C3A	63.38 (17)
C1E—C1—N2—O3	−177.20 (13)	O3—C3A—N14—C1	−26.29 (12)
C1—N2—O3—C3A	−21.41 (15)	C4—C3A—N14—C1	86.69 (13)
N2—O3—C3A—N14	29.51 (12)	C7A—C3A—N14—C1	−141.96 (12)
N2—O3—C3A—C4	−89.57 (12)	O3—C3A—N14—C13A	105.95 (12)
N2—O3—C3A—C7A	153.08 (11)	C4—C3A—N14—C13A	−141.07 (12)
O3—C3A—C4—C40	119.11 (14)	C7A—C3A—N14—C13A	−9.72 (17)
N14—C3A—C4—C40	8.51 (18)	N2—C1—C1E—C2E	−160.17 (16)
C7A—C3A—C4—C40	−126.24 (14)	N14—C1—C1E—C2E	18.8 (2)
O3—C3A—C4—C5	−59.56 (14)	N2—C1—C1E—C6E	20.0 (2)
N14—C3A—C4—C5	−170.17 (11)	N14—C1—C1E—C6E	−161.01 (14)
C7A—C3A—C4—C5	55.08 (14)	C6E—C1E—C2E—C3E	2.1 (3)
C40—C4—C5—N6	132.87 (15)	C1—C1E—C2E—C3E	−177.78 (16)
C3A—C4—C5—N6	−48.55 (16)	C1E—C2E—C3E—C4E	−2.0 (3)
C4—C5—N6—C61	173.08 (13)	C2E—C3E—C4E—C5E	0.3 (3)
C4—C5—N6—C7	50.17 (17)	C2E—C3E—C4E—Cl1	−179.47 (16)
C61—N6—C7—C7A	−179.84 (12)	C3E—C4E—C5E—C6E	1.3 (3)
C5—N6—C7—C7A	−57.44 (16)	Cl1—C4E—C5E—C6E	−178.92 (15)
O3—C3A—C7A—C7	53.94 (13)	C4E—C5E—C6E—C1E	−1.2 (3)
N14—C3A—C7A—C7	166.95 (11)	C2E—C1E—C6E—C5E	−0.4 (2)
C4—C3A—C7A—C7	−60.14 (14)	C1—C1E—C6E—C5E	179.41 (15)
O3—C3A—C7A—C8	174.39 (11)	C5—C4—C40—C41	0.6 (3)
N14—C3A—C7A—C8	−72.60 (15)	C3A—C4—C40—C41	−177.89 (14)
C4—C3A—C7A—C8	60.30 (15)	C4—C40—C41—C46	−141.76 (17)
N6—C7—C7A—C3A	61.13 (14)	C4—C40—C41—C42	39.8 (2)
N6—C7—C7A—C8	−62.13 (14)	C46—C41—C42—C43	1.9 (3)
C3A—C7A—C8—C81	166.54 (12)	C40—C41—C42—C43	−179.61 (17)
C7—C7A—C8—C81	−74.47 (14)	C41—C42—C43—C44	−1.4 (3)
C3A—C7A—C8—S9	41.12 (14)	C42—C43—C44—C45	−0.1 (4)
C7—C7A—C8—S9	160.11 (9)	C43—C44—C45—C46	1.0 (4)
C81—C8—S9—C9A	−85.63 (10)	C44—C45—C46—C41	−0.5 (3)
C7A—C8—S9—C9A	42.81 (11)	C44—C45—C46—Cl2	178.16 (17)
C8—S9—C9A—C10	106.47 (13)	C42—C41—C46—C45	−1.0 (2)
C8—S9—C9A—C13A	−74.58 (12)	C40—C41—C46—C45	−179.52 (16)
C13A—C9A—C10—C11	−3.0 (2)	C42—C41—C46—Cl2	−179.57 (13)
S9—C9A—C10—C11	175.92 (13)	C40—C41—C46—Cl2	1.9 (2)
C9A—C10—C11—C12	1.2 (3)	C7A—C8—C81—C82	−35.1 (2)
C10—C11—C12—C13	2.0 (3)	S9—C8—C81—C82	93.14 (16)
C11—C12—C13—C13A	−3.2 (2)	C7A—C8—C81—C86	149.96 (14)
C12—C13—C13A—C9A	1.3 (2)	S9—C8—C81—C86	−81.81 (16)
C12—C13—C13A—N14	−177.72 (13)	C86—C81—C82—C83	0.9 (3)
C10—C9A—C13A—C13	1.8 (2)	C8—C81—C82—C83	−174.28 (18)
S9—C9A—C13A—C13	−177.20 (11)	C81—C82—C83—C84	−1.0 (3)
C10—C9A—C13A—N14	−179.17 (13)	C82—C83—C84—C85	0.3 (4)
S9—C9A—C13A—N14	1.87 (17)	C83—C84—C85—C86	0.3 (4)
N2—C1—N14—C13A	−118.19 (14)	C84—C85—C86—C81	−0.4 (3)
C1E—C1—N14—C13A	62.74 (18)	C84—C85—C86—Cl3	177.80 (18)
N2—C1—N14—C3A	15.27 (16)	C82—C81—C86—C85	−0.2 (3)
C1E—C1—N14—C3A	−163.80 (13)	C8—C81—C86—C85	175.07 (17)
C13—C13A—N14—C1	7.71 (19)	C82—C81—C86—Cl3	−178.35 (14)
C9A—C13A—N14—C1	−171.33 (12)	C8—C81—C86—Cl3	−3.1 (2)
C13—C13A—N14—C3A	−117.57 (15)		
